# Uniting Mind and Body in Our Health Care and Public Health Systems

**Published:** 2006-03-15

**Authors:** James Lando, Sheree Marshall Williams

**Affiliations:** National Center for Chronic Disease Prevention and Health Promotion, Centers for Disease Control and Prevention; National Center for Chronic Disease Prevention and Health Promotion, Centers for Disease Control and Prevention, Atlanta, Ga

The separation of mental and physical health that exists in our health care and public health systems belies the fact that both exist within individuals in an exquisitely integrated fashion. This issue of *Preventing Chronic Disease* explores that integration. The definition of health provided by the constitution of World Health Organization is unambiguous in this regard: "Health is a state of complete physical, mental, and social well-being and not merely the absence of disease or infirmity" (1). If we are to achieve this goal of complete well-being, we will have to bridge the chasms within our health care and public health systems.

In this issue, we have presented a logic model to display the inputs, activities, and outcomes we expect to occur as the result of a concentrated focus on integrating mental health promotion and mental illness prevention into traditional public health work in chronic disease prevention and health promotion (2). We present the logic model to invite readers of *Preventing Chronic Disease* to help refine it and show others how they might begin conceptualizing and prioritizing such work.

The article by Town et al (3) about health care access among adults who drink excessively is based on data from the Behavioral Risk Factor Surveillance System. The article presents cross-sectional analyses of excessive drinking and health care access and suggests that people who drink excessively are more likely to be uninsured than those who do not. Nevertheless, nearly 80% of excessive alcohol users had been to a health care provider in the last year, providing a critical opportunity for intervention and sensitizing health professionals to the importance of alcohol-related screening and referral. This article and its conclusions confirm the importance of having measures of alcohol use within public health surveillance systems (2).

One of the desired long-term outcomes of our logic model is improved resilience in individuals. Simply put, this means that people should be able to respond appropriately to the types of stress they encounter in their lives so they can function optimally. If we are to achieve this outcome, it seems only logical that we understand the nature of stress and coping in the populations we serve. The qualitative research on African American teens by Chandra and Batada (4) provides interesting insights on stress as perceived by this group of urban Baltimore teens. Although previous research has identified violence as a target for intervention, this study suggests that these ninth graders perceive school pressure and interpersonal relationships to be similarly stressful in their lives. Given the small sample studied, the authors' recommendations for caution in interpretation and for additional research are well advised. Because stress is based on an individual's perception, we need to clearly understand the perspective of the individual and not rely only on external perceptions of environmental factors.

Dementias are both chronic diseases and mental illnesses with significant societal impact and, based on the demographics of aging, can reasonably be predicted to increase in the near future. Although advances are being made in the treatment of dementias, it is unlikely that a cure for this set of diverse diseases will be found anytime soon. As Chapman et al point out (5), destigmatization and the formation of a coherent public health approach to these diseases will be increasingly important in years to come. Surveillance for dementia and research into public health interventions to mitigate its effects are overdue.

Strine et al (6) direct our attention closer to the beginning of the life spectrum. Using nationally representative data from the National Health Interview Survey (NHIS), they characterize elevated emotional and behavioral problems in children diagnosed with attention-deficit/hyperactivity disorder, which was more prevalent in boys, children in low-income households, children with state-sponsored insurance, and children living in households with at most one parent. Nationally representative surveillance systems like NHIS have been shown to be effective and important vehicles for monitoring prevalent mental illnesses.

The sobering article by Colton and Manderscheid (7) shows us that people cared for in the public mental health services system die younger than people in the general population and largely from similar causes. These premature deaths may be in part because of the nature of the mental illness and its treatment, but stigmatization and fragmentation of health care also likely play a role. More research is needed to understand the barriers people with mental illness face in adopting healthy lifestyles and coping with chronic disease. Such research will be critical to designing appropriate interventions to eliminate health disparities among people with mental illness.

Policy development and communication are two central programmatic strategies adopted by the public health community in chronic disease prevention and health promotion, and they figure prominently in our logic model. Interestingly, The Carter Center has undertaken these same strategies to address barriers to care among people with mental illness, many of whom have a chronic mental illness. An article on The Carter Center Mental Health Program (8) describes an impressive set of activities focused on reducing stigma and achieving parity in insurance coverage for mental illness. We would do well to form close collaborations with partners like The Carter Center and to join forces in developing policies and communication strategies that benefit both the mental and physical health of populations. We can claim success when the mental and physical components of our health care and public health systems are as integrated as they are in the people we serve.

This issue of *Preventing Chronic Disease* takes a first step toward developing a coherent strategy to integrate mental and physical health care and public health systems. Achieving this goal will require investments in integrated mental and physical health approaches to surveillance, research, and programmatic activities. Strategic alliances with partners in the mental health field will be critical to our success, as will making the case to public health leaders, policy makers, and the public.

## Author Information

Corresponding Author: James Lando, MD, MPH, National Center for Chronic Disease Prevention and Health Promotion, Centers for Disease Control and Prevention, 4770 Buford Hwy NE, Mail Stop K-40, Atlanta, GA 30341. Telephone: 770-488-5414. E-mail: jlando@cdc.gov.

Author Affiliations: Sheree Marshall Williams, PhD, MSc, National Center for Chronic Disease Prevention and Health Promotion, Centers for Disease Control and Prevention, Atlanta, Ga.
